# Silent echoes: understanding and predicting the widespread mental health impact of mass shootings

**DOI:** 10.3389/fpubh.2026.1889624

**Published:** 2026-08-26

**Authors:** Aadi Mishra, Max E. Coleman

**Affiliations:** 1 Waterford School, Sandy, UT, United States; 2 Department of Sociology, University of Utah, Salt Lake City, UT, United States

**Keywords:** anxiety, collective trauma, depression, gun violence, health inequalities, mass shootings, mediated trauma, public mental health

## Abstract

Half of the 20 deadliest US. mass shootings have occurred in the past decade. We propose and find that the mental health consequences of such shootings are not confined to the immediate community but affect Americans nationwide. We merge two datasets, the Household Pulse Survey and the Northeastern University Mass Killing Database to assess that nationwide effect. Our dataset links 20 consecutive Household Pulse waves (March 2022–November 2023; N ≈ 1,094,000 adults) with every public mass shooting in the same window (17 events). Using survey-weighted logistic regressions that incorporate replicate and person-level weights, we test whether the odds of screening positive for anxiety and depression are higher in survey waves following a public mass shooting compared with waves not preceded by a shooting. We find that mass shootings are associated with increased anxiety and depression after controlling for demographic and socioeconomic variables. Translating effect sizes to counts yields an estimated ~2.27 million additional adults screening positive for anxiety and ~1.29 million additional adults for depression nationwide after a public mass shooting. Moreover, our analyses indicate that higher death counts and younger victims are associated with increased nationwide effects. Analysis of data from survey waves immediately before and after the 2022 Robb Elementary School shooting finds that anxiety and depression increased and that the size of the effect was inversely proportional to geographic distance from the shooting. Therefore, our results indicate that the mental-health costs of mass shootings are widespread, and it is critical to offer public-health responses that extend beyond the immediate communities where mass shootings occur.

The number of public mass shootings has been increasing in the United States in recent decades. According to the (1), 20 percent of the 167 mass shootings over the past 50 years occurred in the last 5 years. More than half happened after 2000, and 33 percent after 2010. Notable examples include the Parkland high school shooting in 2018 (17 killed), the Robb Elementary School shooting in 2022 (21 killed), and the 2017 Las Vegas music festival shooting, the deadliest so far, with 60 killed and 867 injured (2). In 2024, the US. Surgeon General's office issued a 40-page advisory citing gun violence as an urgent public health issue (3).

Mass shootings can adversely affect survivors and their loved ones. In extreme cases, some have died by suicide (4). Broader community impacts include post-traumatic stress

symptoms (PTSS), depression, anxiety, anger, and somatization (5, 6). A study of 44 school shootings found antidepressant use among youth within 5 miles rose by over 20 percent compared to those 10–15 miles away (7). While most research has examined the proximal effects of mass shootings, it is worth asking whether people not directly related to the shooting could also be affected. Although mass shootings account for just 0.5 percent of homicides in the US, they have a disproportionate impact on public perception. A nationwide (8) survey found that over 70 percent of people reported fear of such incidents and hesitancy to visit public spaces such as schools, malls, universities, and movie theaters. Mass shootings increase firearm acquisitions (9). Following the Robb Elementary School shooting, calls surged to a mental health crisis hotline from individuals, directly connected or not, seeking to discuss firearm-related concerns (10). These findings suggest that even those not directly affected, and perhaps living far away, may still experience adverse mental health. Many consider mass shootings frequent, despite their rarity, because of how quickly social media and news outlets spread the news (11).

After a mass shooting, public health officials and policymakers face a challenge: quickly providing mental health support. A major hurdle is identifying who needs help. The traditional focus has been on those in the immediate vicinity. However, if people further away are also affected, they may not receive support because research has not shown they could be similarly impacted. This can delay recovery, as fast and proactive care speeds up treatment (12).

Most research on mass shootings focuses on mental health impacts on local communities, but we test whether mental health impacts extend nationwide. A shooting in Texas may seem irrelevant to someone in New York based on distance alone, yet distance can miss those who experience adverse effects through media exposure, psychological sensitivity, or other factors. We also test whether impacts differ across individuals, since vulnerability to stressors varies (13) and race, sex, or age may shape outcomes. Finally, we test whether shooting characteristics, including victim demographics, predict the severity of mental health effects. Our research has public health implications because shootings are unpredictable and agencies allocate resources in urgent, *ad hoc* ways (6), gaps can lead to further loss of lives (4), and nationwide needs may go unmet under the assumption that impacts are only local.

Drawing on two datasets, the Household Pulse Survey and the Northeastern University Mass Killing Database, we examine the nationwide mental health impact of mass shootings, testing whether effects vary by respondent demographics and shooting characteristics. We find that mass shootings are associated with elevated anxiety and depression nationwide, with effects varying by victim age, casualty count, and victim race, and we conclude with policy implications.

## Background

### Mental health outcomes of mass shootings

Research has demonstrated that people directly affected in mass shootings experience negative mental health consequences such as PTSS, survivor's guilt, depression, and anxiety. Dyb et al. (14) find that post-traumatic stress levels among shooting survivors can be more than six times higher than in the general population. A review of 28 papers found that exposure intensity and severity significantly predict subsequent mental health issues (6). Factors such as proximity to the shooting, hearing gunfire, witnessing the gunman, or being physically close were linked with negative outcomes.

Lowe and Galea (5) reviewed 49 studies and found that only two examined harms beyond the immediate community: Columbine and Virginia Tech. The Columbine study did not measure mental health outcomes, so it cannot speak to nationwide mental health effects (15). The Virginia Tech study found that greater TV viewing of coverage was associated with more stress symptoms among students who were remote from the event (16). Taken together, this meta-analysis shows that there is little research policymakers can rely on to understand how far beyond the immediate community the mental health effects can extend.

In sum, existing research shows that (1) mass shootings adversely affect mental health in the immediate community, but not national impacts; and (2) mental health support is needed after a mass shooting. There is limited research on how shooting characteristics or differential vulnerability shape outcomes. We argue that limited knowledge of impacts beyond the immediate vicinity hampers efficient allocation of resources.

### Exposure and vulnerability to stressors

We first review research in the broader domain of traumatic events to examine whether mental health harms occur only among those directly affected or whether they can be more widespread. Research and policy often suggest that only direct exposure to a mass shooting causes harm. However, studies on events like 9/11 and the Iraq war show that people who were not directly involved but were exposed to media coverage also experience mental health harms (17). A nationwide survey after 9/11 found high stress levels across the country, regardless of location (18). Similarly, after Hurricane Katrina, people with preexisting mental health issues reported greater depression after consuming news about the hurricane, even if they were not personally affected (19). These findings suggest that the impact of traumatic events may extend beyond the event's location.

Recent research describes how traumatic events affect people based on proximity. Semenza and Kravitz-Wirtz (20) propose a three-tier model: (i) direct victims experience the most intense exposure; (ii) secondary witnesses such as family, friends, and first responders are affected through interpersonal ties; and (iii) community members experience chronic stress as gun violence becomes normalized. Building on this model, we propose a fourth tier: media-driven information spread where media exposure to a mass shooting can lead even geographically distant people to experience anxiety or depression.

This fourth tier draws support from scholarship on mediated trauma and cultural meaning-making. Chouliaraki (21) theorizes that media technologies transform distant audiences into spectators of suffering, generating emotional responses that range from detached pity to active solidarity depending on how the suffering is represented. Mass shootings, particularly those involving children or occurring in ostensibly safe public spaces, are represented in ways that collapse psychological distance and invite identification with victims. Alexander (22) further argues that events become culturally traumatic not merely through the harm they inflict on direct victims but through a social process in which the event is collectively interpreted as a threat to group identity and security. A mass shooting can thus function as a cultural trauma: Americans may experience it not as something that happened elsewhere but as evidence that the social contract around public safety has been violated, producing anxiety and depression that extend well beyond the event's geographic footprint.

We draw on research on stress, inequality, and trauma to assess whether mass shooting effects differ across social groups, especially minorities. Pearlin's stress process model (13) argues that stressors accumulate rather than occur in isolation; for example, pandemic-induced job loss may shape how someone reacts to divorce (23). Because exposure is socially patterned, unequal exposure can produce unequal vulnerability (24). Individuals with depression are more likely to forecast negative emotional responses to trauma (25), and those with prior trauma are more likely to experience posttraumatic stress following mass shootings (26).

Racial and ethnic minorities report more frequent exposure to stressors than White counterparts e.g., Black individuals are more likely to live in neighborhoods with high gun violence (27), have firearm injuries requiring inpatient admission that are nine times more common (28), and are nearly 14 times more likely to die from firearm homicide (29). Hispanics experience greater mental health harms due to gun violence (30). However, racial minorities may underreport mental health challenges due to stigma or cultural norms (31) and may become desensitized as frequent trauma is normalized (32). We test two opposing hypotheses: a widely reported mass shooting may worsen Black adults' mental health, or coping and underreporting may hide true impact. We test whether anxiety and depression rise after a mass shooting and whether this varies by race and ethnicity.

We also test relationship between shooting characteristics and media coverage since shootings vary in scale, victim demographics, and media attention. Shootings with child victims, high casualty counts, or socially vulnerable individuals receive more coverage (11). Media may amplify effects through a “cycle of distress” where exposure increases acute stress, which drives more trauma-news consumption and worsens stress responses (33). Conversely news-media could reduce effects through collective coping: official statements, community vigils, and national mourning can reduce emotional isolation (34).

## Data and methods

### Data

To answer these research questions, two datasets were combined that have not previously been used together. The first is the Household Pulse Survey (HPS), launched by the US. Census Bureau in collaboration with five federal agencies including the National Center for Health Statistics (35). The HPS is a publicly accessible deidentified database (see Supplementary Appendix A). Initiated during the COVID-19 pandemic, the survey also tracks changing mental health in the US. population. The same 20-min survey is administered across all 50 states and the District of Columbia. Average weekly sample sizes are around 1,778 respondents per state, with a median of 1,555 (36).

The Household Pulse Survey structure allows an event-linked, repeated cross-sectional design because survey waves were scheduled independently of public mass shootings. The HPS sampling frame and weighting strategy support population-level inference, while the predetermined survey schedule helps address a concern that survey timing was chosen in response to mass shootings. Respondents were contacted by email or text and asked to complete the survey online. Additional details appear in the online supplement.

We then linked HPS data to the Northeastern University Mass Killing Database, compiled by USA TODAY, the Associated Press, and Northeastern University (2). This dataset covers mass shootings in the US. from 2006 to the present (see Supplementary Appendix A). It excludes negligent homicides, such as those from DUI or accidental fires, due to lack of intent. We focus only on public mass shootings, excluding others such as family homicides. The Congressional Research Service (2, 37) defines a public mass shooting as “a multiple homicide incident in which four or more victims are murdered with firearms, not including the offender(s), within one event, and at least some of the murders occurred in a public location or locations in close geographical proximity (e.g., a workplace, school, restaurant, or other public settings), and the murders are not attributable to any other underlying criminal activity or commonplace circumstance (armed robbery, criminal competition, insurance fraud, argument, or romantic triangle).” Hence, we adopted this definition for the purposes of this research.

### Time frame and dataset linking

Although the Household Pulse Survey began in 2020, we wanted to isolate the influence of mass shootings on mental health, so we focused on 2022 and 2023 and avoided the first 2 years of the pandemic. In this period, the first mass shooting was in Buffalo, NY, on May 14, 2022, and the last was in Lewiston, ME, on October 25, 2023. We included 17 public mass shootings. We linked the two datasets by time period. Anxiety (GAD-2) and depression (PHQ-2) were measured in the Household Pulse Survey from March 2022 (Week 44) to November 2023 (Week 63), covering 20 survey waves (Table 1).

**Table 1:** Descriptive statistics and comparison of excluded and analytic observations.

Variables	Data	
	Excluded from analytic sample because of missing outcome or covariate data * N* = 180,691	Used in analysis *N* = 1,094,147
Anxiety		
Anxious	3541 (2.0%)	271,847 (25%)
Not Anxious	177,150 (98%)	822,300 (75%)
Depression		
Depressed	2,520 (1.4%)	199,790 (18%)
Not Depressed	178,171 (99%)	894,357 (82%)
**Household Size (rescaled)**	0.22 (0.18)	0.18 (0.16)
**Age (rescaled)**	0.46 (0.24)	0.49 (0.22)
Race		
White	137,441 (76%)	907,986 (83%)
Black	22,154 (12%)	80,326 (7.3%)
Asian	10,335 (5.7%)	51,489 (4.7%)
Other	10,761 (6.0%)	54,346 (5.0%)
Sex at birth		
Male	76,529 (42%)	471,829 (43%)
Female	104,162 (58%)	622,318 (57%)
Hispanic		
Not hispanic	158,552 (88%)	1,004,453 (92%)
Hispanic	22,139 (12%)	89,694 (8.2%)
Work loss		
Question seen but not answered	8,147 (4.5%)	0 (0%)
Missing data	30,568 (17%)	0 (0%)
Yes	14,801 (8.2%)	92,278 (8.4%)
No	127,175 (70%)	1,001,869 (92%)
Marital status		
Question seen but not answered	9,106 (5.0%)	0 (0%)
Married	91,755 (51%)	628,478 (57%)
Widowed	9,441 (5.2%)	61,473 (5.6%)
Divorced	25,774 (14%)	170,213 (16%)
Separated	3,951 (2.2%)	18,091 (1.7%)
Never married	40,664 (23%)	215,892 (20%)
Household food sufficiency for last 7 days		
Question seen but not answered	9,612 (5.3%)	0 (0%)
Missing data	106,289 (59%)	0 (0%)
Enough of the kinds of food (I/we) wanted to eat	36,258 (20%)	716,626 (65%)
Enough, but not always the kinds of food (I/we) wanted to eat	21,483 (12%)	296,099 (27%)
Sometimes not enough to eat	5,122 (2.8%)	61,236 (5.6%)
Often not enough to eat	1,927 (1.1%)	20,186 (1.8%)

Age and household size were rescaled to a 0–1 range for use in the regression models. For the data used in analysis, the unscaled values are: age, median 53 (IQR 40–66), range 18–89 years; household size, median 2 (IQR 2–3), range 1–10 persons.

While mass shootings have continued beyond this window, we do not extend the analysis into 2024 and 2025 because the Household Pulse Survey underwent substantial changes that compromise comparability. In January 2024, the Census Bureau shifted from biweekly to monthly data collection, altering the temporal resolution on which our design depends. The cross-sectional HPS ended permanently in September 2024 and was replaced in January 2025 by the Household Trends and Outlook Pulse Survey (HTOPS), a longitudinal panel with smaller samples, no state-level estimates, and alternating content that does not administer the GAD-2 and PHQ-2 in every collection period (38). These changes make post-2023 data incomparable with the 20 waves used in this study.

We matched each shooting to the closest Household Pulse Survey period. For example, the Buffalo shooting occurred after the period ending May 9, 2022 and before the period starting June 1, 2022, so we linked it to the June 1 survey. After dropping cases with missing data (180,691 responses, about 14.2%), the analytic dataset had 1,094,147 observations. Missingness was highest for nonresponse to GAD-2 or PHQ-2, followed by loss of work and food sufficiency. Table 1 compares excluded versus analytic data. Anxiety and depression rates in the analytic data track national trends more closely than excluded cases (39). Analytic respondents were slightly older, more likely to be White, and more likely to be married (Table 1). We used survey weights to adjust for complex sample design, nonresponse bias, and related concerns (see “Statistical Analysis”). Since GAD-2 and PHQ-2 ask about symptoms over the prior 2 weeks, the post-shooting wave best captures post-event symptoms when the shooting occurred at least 2 weeks before the survey midpoint. For shootings occurring close to the survey window, the recall period may partially overlap the pre-shooting period, which would likely move estimates toward the null and make our reported associations conservative.

### Measures

#### Outcome variables

The two outcome variables used in the analysis were from the Household Pulse Survey. Anxiety was measured via the Generalized Anxiety Disorder-2 (GAD-2) instrument, while depression was measured through the Patient Health Questionnaire-2 (PHQ-2). Each outcome is binary, indicating whether the respondent met the criteria for anxiety or depression (yes or no) (see Supplementary Appendix B). Research suggests that a threshold of 3 or higher on each two-item screen is acceptable sensitivity and specificity for identifying depressive and anxiety disorders in population samples in the United States (40–42).

#### Predictor variables

We used several predictors from the mass shootings dataset: occurrence of a mass shooting within or prior to the Household Pulse survey period (yes/no), total victims killed, total victims injured, age of victims, victims' sex at birth (% female), victim race and ethnicity (Black, Hispanic, Asian and Pacific Islander, White, or others), distance between the shooting location and the center of the respondent's state (miles; if more than one shooting occurred in a period, we used distance to the most recent shooting), and days elapsed between the shooting and survey completion. Because we did not observe exact survey dates, we used the midpoint of the survey availability period to estimate days elapsed. To measure media coverage intensity, we used Google Trends as a proxy. Although it does not capture content, prior work shows that spikes in search activity closely follow high-intensity media coverage and reflect collective attentiveness to major events (43). When multiple shootings occurred in a short period, we aggregated across shootings, assuming both affected mental health; shooting features (for example, victim counts and victim race) were averaged.

#### Covariates

We controlled for age, race (White, Black, Asian, or other), sex (at birth), ethnicity (Hispanic origin or not), marital status (married, widowed, divorced, separated, never married), household size (total adults and children), employment income loss (respondent or any household member lost employment income), food insufficiency (enough of desired foods, enough but not preferred types, sometimes not enough to eat, often not enough to eat), and week (Household Pulse field period; for example, July 11 to July 27 is Week 47, coded as week = 47).

#### Statistical analysis

The analysis had two parts. First, using 17 mass shootings and 20 Household Pulse surveys, we tested whether the odds of screening positive for nationwide anxiety and depression were higher in survey waves following a public mass shooting compared with waves not preceded by a shooting. Second, we tested whether shooting features (for example, racial or sex-based composition of victims) were associated with different effects. An additional analysis focusing on the Robb Elementary shooting in Uvalde, TX, tests whether effects were nationwide or confined to nearby states (see Part 3).

We used logistic regression because anxiety and depression were the binary outcomes. The data included replicate weights and person-level weights (pweights), so we used survey-weighted logistic regression with a replicate weight design (44), incorporating replicate weights for clustering and stratification and pweights for unequal selection probabilities. We included state fixed effects in Part 1; in Part 2, we dropped state fixed effects after adding distance to the shooting location (measured at the midpoint of each respondent's state). All analyses were conducted in R.

## Results

### Part 1: Nationwide associations between mass shootings and mental health

To answer our research questions, we used a full dataset of 1,094,147 observations and conducted two analyses, one with anxiety and one with depression as the outcome. The predictor was whether a mass shooting occurred since the previous survey (yes or no), along with its interaction with respondents' race. This model included covariates but focused on a single predictor and interaction term; later models examine shooting characteristics. The model tested whether mass shootings were associated with nationwide changes in anxiety and depression after adjusting for potential confounders, and whether effects differed for racial minorities.

This single predictor specification was necessary given the data structure. Across the survey period, there were 11 time periods with mass shootings and 9 without. In periods without shootings, shooting-related variables (for example, total killed or injured) had no variation because they were zero. Distance from the shooting location to the respondent's state was also undefined in no-shooting periods. These variables would be nonzero only when a shooting occurred, creating strong correlation with the occurrence indicator, so we excluded them in this analysis for clarity.

We fit separate models for depression and anxiety. Detailed results are reported in Table 2 (state fixed effects not shown).

**Table 2:** Detailed report of Part 1 (Logistic regression with replicate and person weights).

Variables	Dependent variable	
	Depression log odds (Std errors) odds ratio	Anxiety (Estimate) log odds (Std errors) odds ratio
(Intercept)	−0.249^***^ (0.033) 0.780	0.066^*^ (0.027) 1.068
Predictors		
Shooting occurred: Yes (Reference: No)	0.030^***^ (0.006) 1.030	0.043^***^ (0.005) 1.044
Interactions with race		
Black × Shooting occurred: Yes	0.019 (0.018) 1.019	−0.058^**^ (0.015) 0.944
Asian × Shooting occurred: Yes	0.041 (0.029) 1.042	0.021 (0.021) 1.021
Other × Shooting occurred: Yes	−0.032 (0.022) 0.969	−0.051^*^ (0.016) 0.950
Covariates		
Race (ref: White)		
Black	−0.277^***^ (0.016) 0.758	−0.343^***^ (0.013) 0.710
Asian	−0.311^***^ (0.021) 0.733	−0.494^***^ (0.016) 0.610
Other	0.114^***^ (0.016) 1.121	0.139^***^ (0.015) 1.149
Sex at birth (ref: Male)		
Female	0.029^***^ (0.005) 1.029	0.327^***^ (0.005) 1.387
Ethnicity (ref: Not Hispanic)		
Hispanic	−0.237^***^ (0.008) 0.789	−0.313^***^ (0.007) 0.731
Household size (Rescaled)	−0.160^***^ (0.019) 0.852	−0.153^***^ (0.014) 0.858
Work loss: No (ref: Yes)	−0.518^***^ (0.007) 0.596	−0.556^***^ (0.007) 0.573
Marital status (ref: Married)		
Widowed	0.420^***^ (0.013) 1.522	0.217^***^ (0.011) 1.242
Divorced	0.402^***^ (0.007) 1.495	0.271^***^ (0.008) 1.311
Separated	0.458^***^ (0.019) 1.581	0.326^***^ (0.019) 1.385
Never married	0.438^***^ (0.006) 1.550	0.217^***^ (0.007) 1.242
Week of survey interview	−0.011^***^ (0.0005) 0.989	−0.009^***^ (0.0003) 0.991
Age (Rescaled)	−1.424^***^ (0.015) 0.241	−1.755^***^ (0.014) 0.173
Food sufficiency (ref: Enough of the kinds wanted)		
Enough but not always	1.106^***^ (0.005) 3.022	1.035^***^ (0.005) 2.815
Sometimes not enough	1.786^***^ (0.009) 5.966	1.685^***^ (0.009) 5.392
Often not enough	2.429^***^ (0.016) 11.348	2.393^***^ (0.018) 10.946
Deviance (Null)	1,162,052.264	1,307,626.526
DF (Null)	1,094,146	1,094,146
AIC	1,008,057.152	1,138,257.945
BIC	1,008,938.505	1,139,144.902
Deviance	1,007,951.216	1,138,157.613
Design residual df	9.000	9.000
Number of observations	1,094,147	1,094,147

Odds ratios are the exponentiated log-odds coefficients (e^β^). Statistical significance and standard errors are shown with the log-odds coefficients on the line above; odds ratios are provided as point estimates for interpretability.

Odds ratios for continuous predictors describe the change in odds for a one-unit increase on the scale used in the model.

(1) Because Hispanic ethnicity was modeled as a separate covariate rather than a mutually exclusive racial category, its interaction with shooting is not shown. In a separate model, this interaction was not statistically significant for either depression or anxiety. (2) State fixed effects are omitted for brevity. (3) For the interaction terms, the exponentiated coefficient is a ratio of odds ratios relative to the reference racial group (White) and should not be interpreted as the odds ratio for a mass shooting within that racial group.

^*^p < 0.05;^**^p < 0.01;^***^p < 0.001, based on design-based t-tests.

The results show that mass shootings were associated with increased odds of both depression and anxiety. The coefficient for a mass shooting on depression is 0.03 in log odds (*p* < 0.001), corresponding to an average marginal effect (AME) of 0.42%. For anxiety, the coefficient is 0.043 in log odds (*p* < 0.001), reflecting a 0.59% rise in the predicted probability. Because these are repeated cross-sections, not panel data, we cannot determine whether an individual's risk increased following a mass shooting, only whether the average rate was higher during post-shooting periods.

It is important to note that the interaction between race and the occurrence of mass shootings is not statistically significant in the depression analysis. In the anxiety analysis, we observe that Black and other-race respondents reported a significantly weaker increase in anxiety than White and Asian respondents. Indeed, average marginal effects reveal that the estimated increase in anxiety was not statistically significant for Black respondents or respondents in the “other race” category (see Supplementary Appendix D).

### Limitations of primary analysis and robustness strategy

Although our main analysis uses over one million observations, a limitation is that the primary independent variable is a binary indicator of recent mass shooting exposure that is constant within each Household Pulse Survey wave (a fixed time window for data collection). All respondents in a given wave are either exposed or unexposed. In our dataset, 11 waves occurred immediately after a mass shooting and 9 waves did not. Because exposure varies only across waves, not within them, identification relies on wave-level variation (*n* = 20). This raises concerns about confounding from unobserved time-varying factors, such as national mental health trends or unrelated events, and about overstated precision if the complex survey design is not handled correctly.

We addressed these concerns in three ways. First, we included a time variable (“week”) to adjust for linear change across waves. Second, we incorporated the Household Pulse Survey replicate weights in all models so standard errors reflect the complex survey design. Third, as a robustness check, we interacted mass shooting exposure with marital status, a respondent-level characteristic that varies within waves, to test heterogeneity while recovering some within-wave variation.

In these models, the main effect of mass shooting exposure remained statistically significant and positive, indicating higher symptom levels following mass shootings. The robustness check for marital status (not shown) suggests heterogeneity by social role: married and widowed adults showed stronger associations with post-shooting depression and anxiety, while separated, divorced, and never-married respondents showed weaker associations. These patterns are consistent with differences in social attachment and caregiving responsibility, which may heighten sensitivity to perceived threats against public safety.

### Main findings of Part 1

Our analysis indicates that mass shootings are associated with adverse effects on mental health nationwide, with higher rates of both depression and anxiety. Using data from across the United States, we find that adverse mental health impacts extend beyond the communities where shootings occur. We also examined whether effects differ by race. Past literature suggests that (1) people with prior traumas are more likely to have adverse mental health outcomes after another; (2) racial and ethnic minorities are more likely to experience adverse mental health outcomes associated with gun violence; and (3) racial minorities may underreport mental health challenges due to perceived stigma and cultural differences. In this dataset, Black Americans and people in the “other” racial category (not White, Black, or Asian) report no statistically significant increase in anxiety after a mass shooting. This pattern may reflect desensitization to gun violence among communities with chronic exposure, culturally shaped patterns of symptom reporting, or effective coping strategies developed through repeated adversity. The smaller proportion of Black respondents (7.3% versus 83% White) may also limit detection of very small effects. Why this pattern appears for anxiety, but not depression, warrants further investigation.

### Part 2: Shooting characteristics and mental health outcomes

In this analysis, we examine how characteristics of mass shootings, such as victim demographics, influence mental health outcomes. We created a subset including only weeks when a shooting occurred during the survey period or immediately before the survey began. We used the same covariates as in the prior analysis. Predictors included total victims killed, total victims injured, victim median age, percentage female victims, percentage Black victims, percentage White victims, percentage Hispanic or Latino victims, days since shooting, and distance from the shooting location.

We fit two separate models to estimate effects of shooting features on depression and anxiety. Detailed results are reported in Table 3. Most covariates significantly influenced anxiety and depression outcomes.

**Table 3:** Detailed report of Part 2 (Logistic regression with replicate and person weights).

Variables	Dependent variable	
	Depression log odds (Std error) odds ratio	Anxiety log odds (Std error) odds ratio
(Intercept)	−2.304^***^ (0.243) 0.100	−1.716^***^ (0.224) 0.180
Predictors		
Percentage victims by race		
Hispanic/Latino	0.024^***^ (0.002) 1.024	0.018^***^ (0.002) 1.018
Black	−0.020^***^ (0.002) 0.980	−0.015^***^ (0.002) 0.985
White	0.004^***^ (0.0003) 1.004	0.002^***^ (0.0003) 1.002
Total victims		
Killed	0.014^***^ (0.001) 1.014	0.010^***^ (0.001) 1.010
Injured	0.001^*^ (0.0004) 1.001	−0.000 (0.0004) 1.000
Victim's median age	−0.020^***^ (0.002) 0.980	−0.013^***^ (0.001) 0.987
Percentage victims females	0.004^***^ (0.001) 1.004	0.005^***^ (0.001) 1.005
Days since shooting	0.014^***^ (0.002) 1.014	0.010^***^ (0.002) 1.010
Distance from shooting location	0.000 (0.000) 1.000	−0.000 (0.000) 1.000
Covariates		
Race (ref: White)		
Black	−0.247^***^ (0.014) 0.781	−0.389^***^ (0.011) 0.678
Asian	−0.273^***^ (0.018) 0.761	−0.459^***^ (0.015) 0.632
Other	0.081^***^ (0.014) 1.084	0.092^***^ (0.014) 1.096
Sex at birth (ref: Male)		
Female	0.017^*^ (0.007) 1.017	0.317^***^ (0.007) 1.373
Ethnicity (ref: Not Hispanic)		
Hispanic	−0.209^***^ (0.013) 0.811	−0.270^***^ (0.011) 0.763
Work loss: No (ref: Yes)	−0.513^***^ (0.011) 0.599	−0.566^***^ (0.009) 0.568
Household size (Rescaled)	−0.129^***^ (0.028) 0.879	−0.173^***^ (0.026) 0.841
Food sufficiency (ref: Enough of the kinds wanted)		
Enough but not the kind wanted	1.109^***^ (0.007) 3.031	1.029^***^ (0.006) 2.798
Not always enough	1.793^***^ (0.011) 6.007	1.684^***^ (0.012) 5.387
Often Not Enough	2.421^***^ (0.022) 11.257	2.368^***^ (0.022) 10.676
Marital status (ref: Married)		
Widowed	0.458^***^ (0.017) 1.581	0.203^***^ (0.014) 1.225
Divorced	0.394^***^ (0.010) 1.483	0.252^***^ (0.009) 1.287
Separated	0.378^***^ (0.027) 1.459	0.264^***^ (0.024) 1.302
Never married	0.438^***^ (0.010) 1.550	0.210^***^ (0.009) 1.234
Respondent age (Rescaled)	−1.398^***^ (0.020) 0.247	−1.701^***^ (0.017) 0.183
Week (Survey time)	0.029^***^ (0.005) 1.029	0.026^***^ (0.004) 1.026
Deviance (Null)	645,623.897	726,486.486
DF (null)	606,005	606,005
AIC	560,951.880	634,255.220
BIC	561,235.631	634,543.441
Deviance	560,889.450	634,197.261
Design residual df	54.000	54.000
Number of observations	606,006	606,006

Odds ratios are the exponentiated log-odds coefficients (e^β^). Statistical significance and standard errors are shown with the log-odds coefficients on the line above; odds ratios are provided as point estimates for interpretability.

Odds ratios for continuous predictors describe the change in odds for a one-unit increase on the scale used in the model. Because the percentage-of-victims variables are coded from 0 to 100, their odds ratios describe the effect of a one-percentage-point increase.

^*^ *p* < 0.05;^**^ *p* < 0.01;^***^ *p* < 0.001, based on design-based t-tests.

Several shooting characteristics are significantly associated with mental health outcomes (Table 3). As the number of victims killed increases, the probability of depression increases (probability = 0.195%, log odds = 0.0143, *p* < 0.001), as does the probability of anxiety (probability = 0.162%, log odds = 0.01, *p* < 0.001). As the median age of victims increases, both depression (probability = −0.271%, log odds = −0.01985, *p* < 0.001) and anxiety (probability = −0.222%, log odds = −0.01342, *p* < 0.001) decrease, indicating that when victims are young the mental health impact on respondents is more severe. A higher percentage of female victims is associated with increased depression (probability = 0.0515%, log odds = 0.003778, *p* < 0.001), with a similar pattern for anxiety. More time elapsed since the shooting is associated with higher depression (probability = 0.188%, log odds = 0.01379, *p* < 0.001) and anxiety (probability = 0.171%, log odds = 0.01038, *p* < 0.001), possibly reflecting continued media coverage, social amplification, and rumination. Distance from the shooting location was not statistically significant for either depression or anxiety, consistent with nationwide rather than localized effects.

*Race/Ethnicity of Victims*. As the proportion of White victims increases, the probability of respondents experiencing depression increases (probability = 0.05%, log odds = 0.003668, *p* < 0.001). That is, each additional 1% increase in the proportion of victims that are White increases the respondent's probability of depression by 0.05%. Conversely, a greater share of Black victims is associated with a lower probability of depression (probability = −0.27%, log odds = −0.01979, *p* < 0.001). A similar pattern emerged for anxiety disorders. A higher percentage of White victims killed is associated with a very slight increase in anxiety (probability = 0.0341%, log odds = 0.002068, *p* < 0.001), while a higher percentage of Black victims killed is linked to a decrease in anxiety (probability = −0.244%, log odds = −0.01479, *p* < 0.001). The pattern of results for Hispanic victims was similar to that of White victims: as the percentage of Hispanic victims increases, respondents' anxiety and depression increases.

*Intensity of Media Coverage*. The results of a supplemental analysis show that Google Trends, a proxy for media coverage, was negatively associated with the probability of respondents experiencing depression and anxiety, though the association was only marginally significant for depression (*p* = 0.084) (52). That is, after adjusting for shooting characteristics and individual covariates, an increase in media intensity was linked to a lower probability of reporting symptoms of depression and anxiety. This pattern is consistent with prior work suggesting that extensive media coverage may help mitigate the adverse mental health impact of mass shootings (21, 34) by reducing feelings of helplessness or loss of control and by protecting against psychological distress.

This finding requires further discussion because it appears to contradict the broader argument that media exposure is a pathway through which distant populations experience adverse mental health effects. There are several possible explanations for this pattern. First, Google Trends measures collective search behavior at the event level, not individual media consumption. We cannot determine whether respondents who reported anxiety personally consumed more or less coverage. Second, search behavior may itself be a form of active coping rather than passive exposure. People who actively seek information about a traumatic event may be better equipped to process it (34). Third, high-intensity collective attention may signal institutional acknowledgment and national mourning that reduce emotional isolation. Chouliaraki (21) observes that certain forms of mediated spectatorship can generate prosocial engagement rather than distress, and a similar process may operate after mass shootings. We therefore interpret this result as suggesting that while media may be the vehicle through which distant populations learn of the event, the nature and intensity of collective attention may moderate whether that exposure is harmful or protective.

### Main findings of Part 2

The results indicate that when more people are killed, anxiety and depression increase. When White or Hispanic victims are killed, anxiety and depression increase more than when Black victims are killed. This pattern should be interpreted cautiously. It is consistent with several possible mechanisms, such as, differential media coverage of shootings with White victims (11), normalization of violence against Black Americans, or racialized differences in whose suffering receives national recognition. We acknowledge that the present data cannot distinguish among these mechanisms. Mental health effects are stronger when victims are younger. Effects also increase as more time passes after the shooting, possibly because people have more time to think about it. Distance from the shooting does not predict how strongly people respond, which is consistent with nationwide effects. The effects in this analysis are modest in part because the sample is restricted to weeks when a mass shooting occurred, meaning the baseline level of anxiety and depression is already elevated. This makes the analysis more conservative, so even small changes can be meaningful.^2^

Parts 1 and 2 establish that mass shootings are associated with nationwide increases in anxiety and depression, but the observational design limits causal inference. To strengthen this inference, we conducted a supplementary analysis that examines a specific temporal window around the Robb Elementary shooting in Uvalde, TX and is reported in Part 3 below. This analysis allowed a pre-post comparison while controlling for temporally proximate shootings. Results indicate that depression and anxiety increased following the shooting, with effects that diminished with distance from the shooting location.

### Part 3: The Robb elementary shooting

Studying the impact of mass shootings on mental health poses challenges for causal inference. One of the fundamental issues is the ethical and practical difficulty of manipulating participants' exposure to such traumatic events. Unlike randomized control trials where variables can be carefully controlled, mass shootings are unpredictable and non-replicable events. While experimental approaches are possible, manipulating whether some participants experience a mass shooting (either through a description or a video) has ethical limitations, the outcomes on participants can be harmful and uncontrolled, and such an approach would not capture the true impact as the event was actually unfolding.

Despite these limitations in conducting a causal study on the effects of mass shootings on mental health, an approach can be adopted that comes closer to a causal examination. To understand whether mass shootings have a direct effect on mental health, it is essential to have a clear temporal link between the event (the mass shooting) and the outcome (changes in mental health); it is also crucial to examine whether proximity to the shooting predicts adverse mental health outcomes. The Robb Elementary shooting in Uvalde, TX provided a unique case for such analysis due to its timing. Although this shooting occurred just 10 days after the Tops shooting in Buffalo, NY, and 8 days prior to the Tulsa, OK shooting, no shooting occurred within a month prior to or after these events. The temporally isolated nature of these shootings allowed for a clearer attribution of any observed changes in mental health.

The Robb Elementary shooting took place on May 24, the Buffalo, NY shooting on May 14, and the Tulsa, OK shooting on June 1, 2022. In the analysis that follows, we used the Household Pulse Survey data from two periods: before these shootings (Week 45, April 27–May 9) as the “pre” condition and after the shootings (Week 46, June 1–June 13) as the “post” condition. In this analysis, we used the same covariates as in the prior analyses, controlling for distances from New York and Oklahoma, and focused on the changes in mental health between survey periods. We tested the physical distance between the midpoint of respondents' states and the midpoint of Texas as a predictor in the model. This predictor variable of distance allowed us to test the assumption that only respondents living near a mass shooting are affected while those further away are not affected. This approach allows us to test whether mental-health outcomes shifted between a pre-shooting and post-shooting wave, and whether any shift is moderated by distance from the shooting location. We do not claim causal identification: the design lacks a non-exposed control group.

Two separate analyses were conducted, one with anxiety as the outcome variable and the other with depression. Across both analyses a similar pattern of results emerged. The detailed findings are reported in Table 4.

**Table 4:** Detailed report of Part 3 (Logistic regression with replicate and person weights).

Variables	Dependent variable	
	Depression log odds (Std error) odds ratio	Anxiety log odds (Std error) odds ratio
(Intercept)	−1.552^***^ (0.067) 0.212	−1.034^***^ (0.052) 0.356
Predictors		
Distance from texas (Rescaled)	−0.603^***^ (0.164) 0.547	−0.339^**^ (0.123) 0.712
Condition: Post shooting (Reference: Pre shooting)	0.137^*^ (0.052) 1.147	0.185^***^ (0.044) 1.203
Distance from texas X condition: Post shooting (Reference: Pre Shooting)	−0.256^**^ (0.083) 0.774	−0.160^*^ (0.069) 0.852
Covariates		
Race (ref: White)		
Black	−0.280^***^ (0.040) 0.756	−0.368^***^ (0.036) 0.692
Asian	−0.453^***^ (0.040) 0.636	−0.630^***^ (0.037) 0.533
Others	0.183^***^ (0.036) 1.201	0.107^**^ (0.038) 1.113
Sex at birth: Female (Reference: Male)	0.105^***^ (0.019) 1.111	0.369^***^ (0.017) 1.446
Hispanic: Yes (Reference: No)	−0.261^***^ (0.031) 0.770	−0.256^***^ (0.027) 0.774
Marital status (reference: Married)		
Widow	0.339^***^ (0.036) 1.404	0.070 (0.043) 1.073
Divorced	0.473^***^ (0.025) 1.605	0.308^***^ (0.021) 1.361
Separated	0.452^***^ (0.062) 1.571	0.279^***^ (0.062) 1.322
Never married	0.470^***^ (0.023) 1.600	0.170^***^ (0.022) 1.185
Age (Rescaled)	−1.520^***^ (0.053) 0.219	−1.931^***^ (0.040) 0.145
Household size (Rescaled)	−0.166^**^ (0.055) 0.847	−0.132^*^ (0.056) 0.876
Food sufficiency (ref: Enough of the kinds wanted)		
Enough but not the kind wanted	1.165^***^ (0.018) 3.206	1.098^***^ (0.016) 2.998
Not always enough	1.859^***^ (0.035) 6.417	1.702^***^ (0.036) 5.485
Often not enough	2.520^***^ (0.059) 12.429	2.610^***^ (0.052) 13.599
Distance from New York (Rescaled)	0.090^*^ (0.042) 1.094	0.042 (0.037) 1.043
Distance from oklahoma (Rescaled)	0.763^***^ (0.135) 2.145	0.510^***^ (0.110) 1.665
Deviance (Null)	113,506.936	126,639.769
DF (Null)	107,020	107,020
AIC	98,041.936	109,836.856
BIC	98,219.255	110,017.767
Deviance	97,987.639	109,786.151
Design residual df	60.000	60.000
Number of observations	107,021	107,021

Odds ratios are the exponentiated log-odds coefficients (e^β^). Statistical significance and standard errors are shown with the log-odds coefficients on the line above; odds ratios are provided as point estimates for interpretability.

Odds ratios for continuous predictors describe the change in odds for a one-unit increase on the scale used in the model. Distance variables are rescaled to a 0–1 range.

For the distance × condition interaction term, the exponentiated coefficient is a ratio of odds ratios and should not be interpreted as a standalone odds ratio; it describes how the effect of the post-shooting condition changes with distance from the shooting location.

^*^p < 0.05;^**^ *p* < 0.01;^***^ *p* < 0.001, based on design-based t-tests.

**Figure 1:**
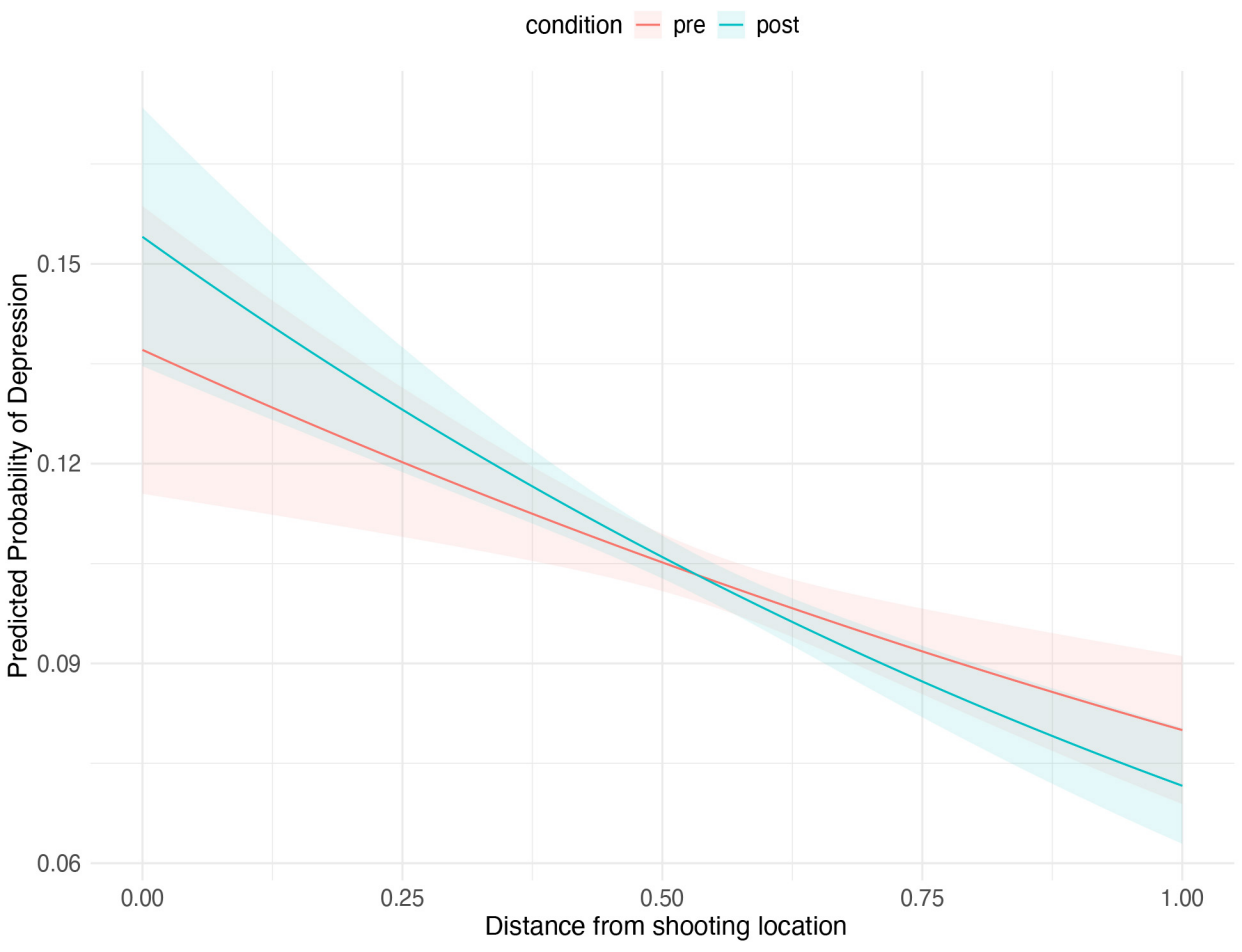
Interaction between pre- vs. post-shooting condition and distance (Depression). The estimated effect of the shooting at any given distance is the vertical gap between the post-shooting and pre-shooting lines. The gap is positive near the shooting location and narrows with distance, consistent with a stronger mental-health impact among respondents living closer to the event. Estimates at the nearest and farthest distances are based on fewer respondents and are correspondingly less precise, so the apparent crossover at the farthest distances should not be interpreted as a substantively reversed effect.

Depression analysis. The odds of experiencing depression were higher in the post-shooting period than in the pre-shooting condition (log odds = 0.137, *p* < 0.011). The interaction between shooting condition and distance was also significant (log odds = −0.256, *p* < 0.01), indicating that the increase in depression following a mass shooting is smaller for people located farther from the event. Thus, the results do not suggest that distance reduces depression on its own, but rather that the increase in depression following a mass shooting is attenuated with greater distance. Figure 1 depicts this interaction. The x-axis is the rescaled distance from the shooting location to the center of each respondent's state's geographic center. For instance, Arkansas has a rescaled distance of 0.22, Minnesota 0.55, Delaware 0.75, and Alaska 1.

**Figure 2:**
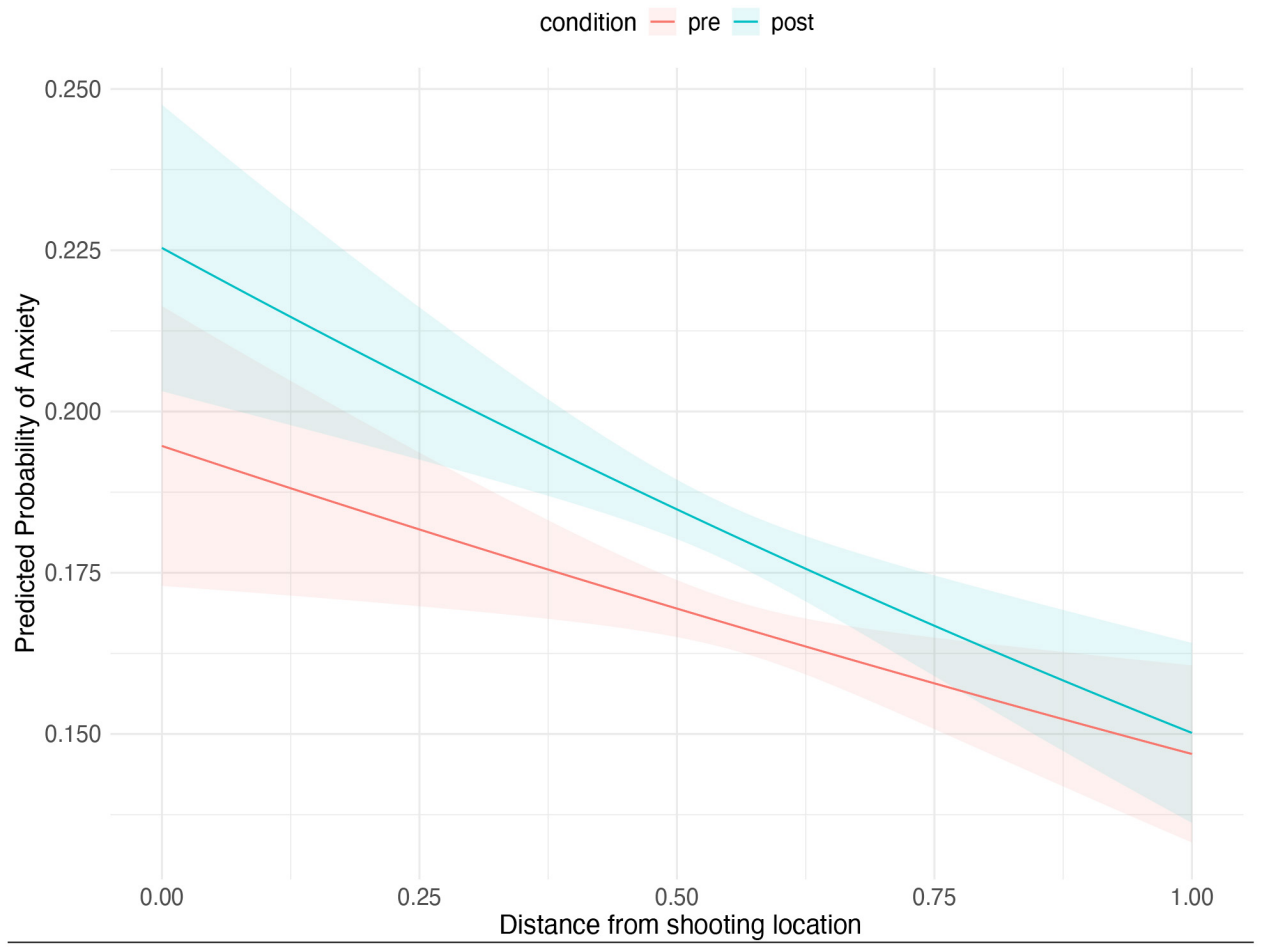
Interaction between pre- vs. post-shooting condition and distance (Anxiety). The estimated effect of the shooting at any given distance is the vertical gap between the post-shooting and pre-shooting lines. The post-shooting line remains above the pre-shooting line across the plotted range, though the gap narrows with distance, indicating that the increase in anxiety was larger closer to the shooting.

Anxiety analysis. Similar to the depression analysis, the odds of experiencing anxiety were higher in the post-shooting period compared to the pre-shooting condition (log odds = 0.185, *p* < 0.01). The interaction between pre- vs. post-shooting condition and distance is statistically significant (coefficient = −0.159, *p* < 0.03). Figure 2 depicts this interaction.

### Main findings of Part 3

The analyses indicated an increase in depression and anxiety in the Robb Elementary shooting. This provides evidence of the association of mass shootings on mental health. Importantly, it was observed that this effect was inversely proportional to the distance from the shooting location, indicating that geographic distance can moderate the impact of mass shootings on mental health outcomes: with the estimated post-shooting increase attenuating as distance from the shooting increases.

## Discussion

Across analyses, mass shootings are associated with higher rates of depression and anxiety nationwide, including among adults not directly affected, challenging the view that impacts are limited to the immediate vicinity.

The findings matter sociologically because they suggest that the health burden of mass shootings is produced not only through direct exposure to violence but also through social processes that make distant violence visible, emotionally salient, and unequally recognized.

Translating the Part 1 coefficients to population counts (detailed calculations are reported in Supplementary Appendix C), we estimate that after a public mass shooting the number of adults in the US. experiencing depression increases by an additional 1.29 million and those experiencing anxiety by an additional 2.27 million. These are large population-level estimates indicating that the impact of mass shootings is not confined to those directly affected but is experienced nationwide. These model-based counts should be interpreted as estimated additional adults screening positive on brief symptom measures, not as additional clinical diagnoses. The analysis of the Robb Elementary shooting (see Part 3) further shows that depression and anxiety increased in the post-shooting period, with effects diminishing with distance from the shooting location, providing additional evidence that effects extend beyond the immediate community while also being moderated by geographic proximity. These counts represent estimated additional individuals screening positive within the same survey wave. However, given the cross-sectional nature of the publicly available dataset that we accessed for the analysis, we are not able to test for the persistence of these symptoms beyond the immediate post-shooting period.

We also test whether effects differ by race. While two research streams make opposing predictions, our analyses do not indicate that racial minorities are disproportionately affected. Black and other-race Americans report smaller changes in anxiety than other racial groups, which could be consistent with coping strategies developed through chronic exposure to gun violence, including community-based support networks and emotional regulation developed through repeated adversity, as well as culturally shaped patterns of symptom underreporting (31). We do not attribute these patterns to insufficient psychosocial resources among Black and Hispanic Americans; rather, we focus on differences in prior stressor exposure [see (45–48)]. Mental health outcomes vary with shooting characteristics. As the number of victims increases, as the median age of victims decreases, as the percentage of female victims increases, and as the time between the shooting and data collection increases, depression and anxiety rise more. Respondents report greater anxiety and depression when White or Hispanic victims are killed than when Black victims are killed, a pattern that may reflect differential media coverage that affords greater prominence to shootings with White victims (11), normalization of violence against Black Americans, or a broader empathy gap in which the suffering of Black victims is accorded less national significance. Our data cannot adjudicate among these mechanisms, and future research should investigate their relative contributions.

These findings indicate a need for mental health support that is not confined to the shooting location. Because shootings are unpredictable, providers cannot rely on geography to infer where support is needed. Our results are consistent with a nationwide approach, including a widely publicized helpline and targeted outreach to vulnerable populations. Prior work argues for removing barriers to help-seeking as a timely and proactive intervention strategy (12). Evidence from Finland indicates that over half of students with high trauma-related symptoms after a school shooting found immediate support beneficial (49), consistent with the view that timely psychosocial support can reduce post-traumatic stress.

Unequal help-seeking is a constraint. Many minoritized groups do not seek help despite mental health challenges because of anticipated stigma (31), so timely, accessible, and low-barrier support remains essential.

The negative Google Trends coefficient captures collective search behavior at the event level, not individual exposure to distressing content. The mechanism described here concerns individual exposure to graphic or repetitive imagery on social platforms, which may operate differently from aggregate search activity. Our results are also consistent with the idea that media exposure may contribute to increased risk of anxiety or depression among Americans far from the event. Viral video exposure to other traumatic events has been linked to increased depression and PTSS in Black Americans (50), and a similar pattern may be occurring with mass shootings. This concern is amplified by platforms such as Instagram and TikTok where sensational imagery can be algorithmically prioritized.

### Limitations and future directions

We acknowledge that the best way to estimate the true nationwide impact of a mass shooting is to measure mental health right before and after the event, but shootings are random and unpredictable, so that study-design is hard to time and expensive. Because retrospective recall is also limited, combining the Household Pulse Survey with the Mass Killing Database allows a practical way to study the nationwide mental health impact of such rare and unfortunate events.

We also acknowledge that the presence of anxiety and depression are based on self-reported scale measures rather than clinical diagnoses. A second limitation is that we estimate group-level change rather than individual change over time because the Household Pulse Survey does not provide sufficient panel data; repeat participation is limited and anonymization prevents tracking. Another concern is that national estimates could be driven by states where shootings occurred. We addressed this by excluding those states during shooting periods; the sensitivity analysis still included over 1,063,373 responses from states not affected by any shootings and showed consistent increases in depression and anxiety.

Moreover, Black Americans report smaller changes in anxiety than White Americans, which could reflect underreporting, desensitization to gun violence, or other mechanisms. This pattern is consistent with research indicating that PHQ-measured depressive symptoms have been found to be lower among Black than White adults in national data (51); thus, the lower post-shooting symptom levels observed among Black respondents may be shaped by differences at baseline. Black victims elicit weaker adverse mental health outcomes than White or Hispanic victims, a pattern consistent with a racial empathy gap, though differential media coverage and chronic violence exposure remain plausible alternative explanations. Future work should also compare gun owners and non-owners.

Gun violence is an increasing problem in the United States, and mass shootings in particular have risen sharply in recent decades. The present study adds that their mental-health consequences may extend beyond the communities where shootings occur. Public-health responses after mass shootings should consider needs beyond the immediate community.

A limitation is that distance is measured from the center of the respondent's state to the shooting site, since this is the smallest geography available in the Household Pulse Survey public-use files. However, one could argue that this does not capture within-state differences. For example, respondents in El Paso and Houston are coded the same even though they are about 550 and 250 miles from Uvalde, respectively. Similarly, a respondent in New York City is much farther from the Buffalo shooting than a respondent in Pittsburgh, but our analysis may not be able to capture this. Finally, Google Trends captures overall search activity, not individual media use, coverage content, or exposure on platforms like TikTok or Instagram. Other measures, such as population mobility, were outside the scope of this data linkage but could be examined in future research.

## Data Availability

Publicly available datasets were analyzed in this study. Household Pulse Survey data are available from the U.S. Census Bureau at https://www.census.gov/programs-surveys/household-pulse-survey/data/datasets.html. Mass shooting data were obtained from the Associated Press/USA TODAY/Northeastern University Mass Killing Database at https://cssh.northeastern.edu/sccj/mass-killing-database/. Google Trends data were obtained from Google Trends at https://trends.google.com. The R code used to estimate the models is available on request.
