# Supplementary material for: Silent echoes: understanding and predicting the widespread mental health impact of mass shootings
Source: Front Public Health. 2026 Aug 26;14:1889624. doi: 10.3389/fpubh.2026.1889624 (PMC13560487; doi:10.3389/fpubh.2026.1889624)
Supplement: Supplementary file 1 [file Data_Sheet_1.pdf]

Online Supplementary Material

Appendix A: Data Description, Linking Procedure, Model Variable Description

Datasets

The U.S. Census Bureau's Household Pulse Survey, a publicly accessible resource, measured Americans' mental health during and after the COVID-19 pandemic through monthly surveys. We linked this dataset to a dataset on public mass shootings in the US as compiled by Northeastern University's Mass Killing Database. By analyzing anxiety and depression levels reported in the Household Pulse Survey in the time period following mass shootings, we sought to examine the nationwide mental health impact of mass shootings.

Household Pulse Survey Data

The Household Pulse Survey (HPS) is an initiative launched by the U.S. Census Bureau in collaboration with five federal agencies including the National Center for Health Statistics (National Center for Health Statistics, 2023). It started as an effort to capture the social and economic impacts of the COVID-19 pandemic on American households. This survey, which started on April 23, 2020, has progressed through multiple phases, with Phase 4.0 being conducted in 2024. The same 20-minute survey is administered in all 50 states plus the District of Columbia (DC). Sample sizes average around 1778 respondents per state per week, with a median of 1555 (Sun et al, 2022).

Households are selected randomly based on their email addresses and cell phone numbers linked to the Census Bureau Master Address File (MAF). One respondent per household is invited to participate via email or text message, and the data collection is continuous throughout each phase of the survey. This survey covers a broad range of topics including employment status, consumer spending, food security, housing, education disruptions, physical and mental

wellness, access to health care, and health insurance coverage (SHADAC, 2021). Specific to mental health, the Household Pulse Survey measures the frequency of symptoms related to anxiety and depression using Generalized Anxiety Disorder-2 (GAD-2) and the Patient Health Questionnaire-2 (PHQ-2). In summary, the Household Pulse Survey provides critical insights into the socio-economic and mental health status of U.S. households (U.S. Census Bureau, 2024).

Mass Shooting Dataset

The mass killing database is compiled by USA TODAY, the Associated Press, and Northeastern University (USA TODAY, 2022). It contains information on mass killings in the United States from 2006 to the present. A mass killing is defined as the intentional killing of four or more victims within a 24-hour period by any means. Importantly, the database excludes negligent homicides, such as those related to driving under the influence or accidental fires, due to the absence of intent.

The data is compiled using the FBI's Supplementary Homicide Reports (SHR) to identify potential incidents, along with media accounts, court documents, academic journal articles, and local law enforcement records. The database contains data tables detailing incidents, offenders, victims, and weapons used. We limit our analyses to *public* mass shootings (see Note 1 in the original manuscript).

Time Frame and Datasets Linking

To avoid limiting the generalizability of our findings, we excluded data from the first two years of the pandemic (2020 and 2021), focusing instead on public mass shootings that occurred in 2022 and 2023. In our data the first mass shooting of this period was in Buffalo, NY, on May 14, 2022, and the last was in Lewiston, ME, on October 25, 2023. In total, we included 17 public

mass shootings in our analysis. Alongside this, we used data from the Household Pulse Survey, covering responses from March 2022 (Pulse data Week 44) to November 2023 (Pulse data Week 63), which consisted of 20 distinct Pulse surveys.

We linked these two sets of data since they both have a time aspect. The timing of the mass shootings was matched with the closest Household Pulse Survey periods. For example, the Buffalo shooting happened between the survey collection periods that ended on May 9, 2022, and the next that started on June 1, 2022. Therefore, we associated it with the data from the June 1st survey. This method helped ensure that the survey data reflected the mental health impact of the shootings as accurately as possible. Overall, this approach resulted in a dataset of 1,087,286 data points. This dataset allowed us to examine how public mass shootings affect mental health.

Appendix B: Anxiety and Depression Scales

Generalized Anxiety Disorder-2 (GAD-2)

Over the last 2 weeks, how often have you been bothered by the following problems?

Q1 Feeling nervous, anxious or on edge:

Not at all (0), Several days (1), More than half the days (2), Nearly every day (3)

Q2 Not being able to stop or control worrying

Not at all (0), Several days (1), More than half the days (2), Nearly every day (3)

A total score of 3 or higher across these two items indicates a risk for generalized anxiety disorder (National Center for Health Statistics, 2023). The outcome variable, anxiety, is a binary variable, indicating whether the respondent had generalized anxiety disorder (yes or no).

Patient Health Questionnaire-2 (PHQ-2)

This instrument involves two questions that assess the frequency of depressive symptoms experienced by the respondent in the past two weeks:

1. How often have you had little interest or pleasure in doing things?
2. How often have you felt down, depressed, or hopeless?

Respondents rate each of these items on a scale ranging from 'Not at all' (0) to 'Nearly every day' (3). A total score of 3 or higher on these items indicates risk for depressive disorder (National Center for Health Statistics, 2023). The outcome variable for depression, similar to that for anxiety, is a binary variable—whether the respondent had a depressive disorder (yes or no)

Appendix C: Calculating the True Impact of Mass Shootings

Calculating additional number of people experiencing depression:

1. **Baseline Odds for Depression:**
   - On an average 22.8% US adults reported depression between March 2022 (Pulse data Week 44) to November 2023 (Pulse data Week 63). [1]
   - The formula to convert a proportion (*P)* to odds is Odds=*P*/1-*P*
   - Therefore, Baseline Odds = 0.228/(1-0.228)= 0.2953. This represents the odds of a person experiencing depression without any mass shooting.
2. **Increased Odds After Applying the Odds Ratio:**
   - Given log odds of 0.029, the Odds Ratio = $e^{0.029} \approx 1.029$
   - Therefore, Increased Odds = Baseline Odds × Odds Ratio = 0.2953 × 1.029 = 0.3039
   - After the mass shooting, the odds of experiencing depression increase to 0.3039.
3. **Increased Proportion of Depression:**
   - To convert odds back to a proportion, the formula is Proportion = Odds/(1+Odds)
   - Increased Proportion = 0.3039/(1+0.3039) = 23.30%. This is the proportion of the population that would experience depression after the mass shooting, reflecting the increase due to the event.
4. **Estimating the Number of Additional People Experiencing Depression:**
   - The increase in the number of people is the difference between the increased proportion and the baseline proportion, multiplied by the U.S. adult population.

[1] Baseline proportion was obtained from: https://www.cdc.gov/nchs/covid19/pulse/mental-health.htm. This is calculated by taking an average level of anxiety and depression during all of the waves of the survey that were used in the analysis.

- ▶ Additional People = U.S. Adult Population × (Increased Proportion - Baseline Proportion) = 258 million x (0.2330-0.228) = 1,290,000 (rounded)
- ▶ This is the additional number of people estimated of experiencing depression following a mass shooting, over and above the baseline number.

In summary, due to the mass shooting, the number of people experiencing depression is estimated to increase by an additional 1.29 million people.

Calculating additional number of people experiencing anxiety:

1. **Baseline Odds for Anxiety:**

   On an average 29.3% US adults reported anxiety[2] between March 2022 (Pulse data Week 44) to November 2023 (Pulse data Week 63). Therefore, Baseline Odds = 0.293/(1−0.293) = 0.4144 (rounded). This represents the baseline odds of a person experiencing anxiety.

2. **Increased Odds After Applying the Odds Ratio:**
   - ▶ Given log odds of 0.042, the Odds Ratio = $e^{0.042} \approx 1.043$
   - ▶ Increased Odds = 0.4144×1.043 ≈ 0.4322.

3. **Increased Proportion of Experiencing Anxiety:**
   - ▶ Increased Proportion = 0.4322/(1+0.4322) ≈0.3018 or 30.2% (rounded). This is the proportion of the population that would experience anxiety after the mass shooting.

4. **Estimating the Number of Additional People Experiencing Anxiety:**
   - ▶ Additional People = 258 million (0.3018−0.293) = 2,270,400 (rounded). This is the additional number of people estimated of experiencing anxiety following a mass shooting.

[2] Baseline proportion was obtained from: https://www.cdc.gov/nchs/covid19/pulse/mental-health.htm . This is calculated by taking an average level of anxiety and depression during all of the waves of the survey that were used in the analysis.

In summary, due to the mass shooting, the number of people experiencing anxiety is estimated to increase by an additional 2.27 million people.

APPENDIX D: Racial Differences in the Effects of a Mass Shooting:

Average Marginal Effects (Based on Table 2)

Table S1. Average Marginal Effects of Mass Shooting on Depression

Race	Term	Contrast	Estimate	Std. Error	*z*	*Pr(>|z|)*
White	shooting occurred	Yes - No	0.0037	0.000815	4.54	<0.001
Black	shooting occurred	Yes - No	0.00699	0.00252	2.78	0.00543
Asian	shooting occurred	Yes - No	0.00805	0.00327	2.46	0.014
Any other race alone or in combination	shooting occurred	Yes - No	-0.000737	0.00332	-0.222	0.824

Table S2. Average Marginal Effects of Mass Shooting on Anxiety

Race	Term	Contrast	Estimate	Std. Error	z	Pr(>|z|)
White	shooting occurred	Yes - No	0.00647	0.000784	8.26	<0.001
Black	shooting occurred	Yes - No	-0.00269	0.00247	-1.09	0.277
Asian	shooting occurred	Yes - No	0.00827	0.0028	2.96	0.00311
Any other race alone or in combination	shooting occurred	Yes - No	-0.0023	0.00302	-0.76	0.447

REFERENCES

National Center for Health Statistics. (2023). *Anxiety and depression: Household Pulse Survey – COVID-19*. Centers for Disease Control and Prevention. Retrieved November 21, 2024, from https://www.cdc.gov/nchs/covid19/pulse/mental-health.htm

SHADAC. (2021). *Measuring Coronavirus Impacts with the Census Bureau's New Household Pulse Survey: Utilizing the data and understanding the methodology*. State Health Access Data Assistance Center. Retrieved November 21, 2024, from https://www.shadac.org/Household-Pulse-SurveyMethods

Sun, A., Parker, P. A., & Holan, S. H. (2022). Analysis of household pulse survey public-use microdata via unit-level models for informative sampling. *Stats, 5*(1), 139–153.

U.S. Census Bureau. (2024). *Household Pulse Survey: Measuring emergent social and economic matters facing U.S. households*. Retrieved November 21, 2024, from https://www.census.gov/data/experimental-data-products/household-pulse-survey.html

USA TODAY. (2022, August 18). Data: Mass killings database reveals trends, details, and anguish in every US event since 2006. *The Associated Press, Northeastern University, and USA TODAY*. Retrieved from https://www.usatoday.com/in-depth/graphics/2022/08/18/mass-killings-database-us-events-since-2006/9705311002/
